# A Novel Variant in *TPM3* Causing Muscle Weakness and Concomitant Hypercontractile Phenotype

**DOI:** 10.3390/ijms242216147

**Published:** 2023-11-09

**Authors:** Katarzyna Robaszkiewicz, Małgorzata Siatkowska, Renske I. Wadman, Erik-Jan Kamsteeg, Zhiyong Chen, Ashirwad Merve, Matthew Parton, Enrico Bugiardini, Charlotte de Bie, Joanna Moraczewska

**Affiliations:** 1 Department of Biochemistry and Cell Biology, Kazimierz Wielki University, 85-671 Bydgoszcz, Poland; robkat@ukw.edu.pl (K.R.); gosia.sl@ukw.edu.pl (M.S.); 2 Department of Neurology, UMC Utrecht Brain Center, University Medical Center Utrecht, Utrecht University, 3584 CX Utrecht, The Netherlands; r.i.wadman@umcutrecht.nl; 3 Department of Genetics, Radboud University Medical Center, 6525 GA Nijmegen, The Netherlands; erik-jan.kamsteeg@radboudumc.nl; 4 Department of Neuromuscular Disease, UCL Queen Square Institute of Neurology, The National Hospital for Neurology, London WC1N 3BG, UK; zhiyong.chen@nhs.net (Z.C.); matt.parton@nhs.net (M.P.); e.bugardini@ucl.ac.uk (E.B.); 5 Department of Neurology, National Neuroscience Institute, Singapore 308433, Singapore; 6 Department of Neuropathology, UCL Queen Square Institute of Neurology, The National Hospital for Neurology, London WC1N 3BG, UK; amerve@nhs.not; 7 Department of Genetics, University Medical Utrecht, 3584 CX Utrecht, The Netherlands; c.i.debie-2@umcutrecht.nl

**Keywords:** nemaline myopathy, tropomyosin, pathogenic variant, thin filament

## Abstract

A novel variant of unknown significance c.8A > G (p.Glu3Gly) in *TPM3* was detected in two unrelated families. *TPM3* encodes the transcript variant Tpm3.12 (NM_152263.4), the tropomyosin isoform specifically expressed in slow skeletal muscle fibers. The patients presented with slowly progressive muscle weakness associated with Achilles tendon contractures of early childhood onset. Histopathology revealed features consistent with a nemaline rod myopathy. Biochemical in vitro assays performed with reconstituted thin filaments revealed defects in the assembly of the thin filament and regulation of actin–myosin interactions. The substitution p.Glu3Gly increased polymerization of Tpm3.12, but did not significantly change its affinity to actin alone. Affinity of Tpm3.12 to actin in the presence of troponin ± Ca^2+^ was decreased by the mutation, which was due to reduced interactions with troponin. Altered molecular interactions affected Ca^2+^-dependent regulation of the thin filament interactions with myosin, resulting in increased Ca^2+^ sensitivity and decreased relaxation of the actin-activated myosin ATPase activity. The hypercontractile molecular phenotype probably explains the distal joint contractions observed in the patients, but additional research is needed to explain the relatively mild severity of the contractures. The slowly progressive muscle weakness is most likely caused by the lack of relaxation and prolonged contractions which cause muscle wasting. This work provides evidence for the pathogenicity of the *TPM3* c.8A > G variant, which allows for its classification as (likely) pathogenic.

## 1. Introduction

Congenital myopathies are a heterogeneous group of neuromuscular disorders mostly linked to mutations in genes encoding skeletal muscle proteins. So far, mutations in about 50 different genes have been identified, indicating that congenital myopathies result from various functional defects in proteins that are essential for skeletal muscle structure, development, contraction, and protein turnover [1,2,3].

The heterogeneous nature of congenital myopathies is manifested not only by the involvement of different genes, but also by the diversity and severity of the clinical presentation and histopathology of muscle fibers in patients with mutations in the same genes. The most common symptom of congenital myopathy is progressive muscle weakness, which affects overall mobility, facial expression, bulbar function, and respiratory function. In some patients, muscle weakness coexists with joint contractures at different stages within the natural history of individual diseases [4,5,6]. Histopathological features can overlap extensively between the causes of congenital myopathy [4,7]. Clinically distinguishing between the different types of congenital myopathies can be difficult.

Pathogenic variants in *TPM3* (OMIM 191030), the gene encoding the tropomyosin isoform Tpm3.12 expressed in type 1 slow twitch muscle fibers, have been associated with nemaline myopathy, cap myopathy, and congenital fiber type disproportion [1,2,8]. Until now, 16 inherited or de novo pathogenic variants have been reported in *TPM3* [1,7,9,10]. The variants result in amino acid substitutions distributed throughout the length of *TPM3* gene, thereby impairing the function of Tpm3.12 [8].

Tropomyosins (Tpms) form a large family of regulatory proteins that bind to actin filaments to stabilize and control the filaments’ interactions with many binding proteins. All members of the tropomyosin family are dimeric α-helical proteins folding into coiled-coil rod-like structures. Due to its ability to overlap end-to-end, Tpm molecules polymerize along each strand of actin as uninterrupted chains [11]. In striated muscle, Tpm forms a Ca^2+^-dependent regulatory complex with troponin (Tn), a protein which contains three subunits—the Ca^2+^-binding TnC, inhibitory TnI, and Tpm-binding TnT. The central segment of Tpm interacts with the core domain of Tn assembled from TnC, TnI, and the C-terminal half of TnT. The N-terminal half of TnT (TnT1) extends from the core domain towards the end-to-end overlap region of the tropomyosin chain associated with the opposite long-pitch helix of actin [12,13,14]. This complex molecular assembly regulates contraction by controlling interactions between actin and myosin heads in a Ca^2+^-dependent manner via steric blocking and cooperative mechanisms. As activation and relaxation of contraction in striated muscle requires multi-subunit cooperation within the thin filament, it is not surprising that mutations in most proteins of the thin filament disrupt regulation of actin–myosin interactions.

In this paper, we describe the clinical and biochemical effects of the novel missense variant c.8A > G (p.Glu3Gly) in exon one of *TPM3*. The mutation was detected in two unrelated families, and resulted in autosomal dominant congenital nemaline myopathy with slowly progressive muscle weakness and Achilles tendon contractures. This mutation is associated with a molecular hypercontractile phenotype, defined based on the altered polymerization of Tpm3.12, its interaction with actin and TnT, and defects in Tpm-dependent regulation of actin–myosin interactions.

## 2. Results

### 2.1. Case Series Report

Patient 1 is a 30-year-old Caucasian male who first visited the outpatient neurology clinic at the age of 17 years. He experienced normal motor development (walking at 11 months, able to run, cycling at 3.5 years). From the age of 10 years, slowly progressive muscle weakness of his foot dorsiflexion with Achilles tendon contractures were noticed. This resulted in difficulties with running, climbing stairs and rising from a kneeling position. By the age of 18 years, he also experienced weakness in handgrip strength and difficulty lifting his arms. There were no symptoms suggestive of nocturnal hypoventilation or cardiac dysfunction.

Neurologic examination at the age of 19 revealed a slender posture with mild myopathic facies, high arched palate, pes cavus, severe atrophy of the upper legs, and contractures of both Achilles tendons. Muscle strength examination revealed symmetric muscle weakness of supraspinatus (MRC grade 4), deltoid (MRC grade 4), gluteal (MRC grade 4), peroneal (MRC grade 4), and feet dorsiflexors (MRC grade 4) (all other muscles were MRC 5). Tendon reflexes were absent. On follow-up examination, muscle strength in foot dorsiflexors (MRC grade 3) deteriorated. This was accompanied with the progression in severity of bilateral Achilles tendon contractures.

Echocardiography revealed a slightly decreased left ventricular systolic function with an ejection fraction of 47–53%. Forced vital capacity was 5.94 L (97% of predicted) while forced expiratory volume in 1 s (FEV1) was 3.90 L.

**Figure 1:**
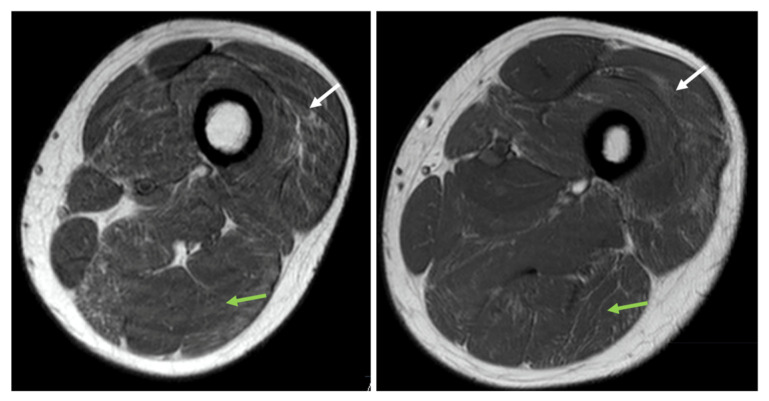
MRI T1 axial view of the left upper leg at two levels. There is fatty infiltration of the vastus lateralis (white arrow) and biceps femoris (green arrow), most pronounced in the vastus lateralis and biceps femoris compartments of these muscles.

Serum creatine kinase (CK) activity was increased (range 1000–1200 U/L; reference range: 40–320 IU/L), and had remained stable over the course of the patient’s follow-up. Nerve conduction studies showed normal motor and sensory conduction velocities. Electromyography showed polyphasic, normal duration motor unit action potentials (MUAPs), without spontaneous activities. Muscle MRI revealed symmetric fatty replacement in upper and lower leg muscles (Figure 1), as well as in the triceps and deltoids. Histopathological analysis of the muscle biopsy from vastus lateralis muscle showed myopathic features associated with the presence of nemaline rod-like structures.

A whole exome sequencing (WES)-based myopathy gene panel identified a heterozygous c.8A > G (p.(Glu3Gly)) variant of unknown significance in the *TPM3* gene. Analysis of the rest of the exome did not identify other pathogenic causative variants.

Patient 2 was the mother of Patient 1. She reported normal motor development until the age of 3–4 years, when her parents noticed that she had developed difficulties with running and was falling frequently. At the age of 16 years, she underwent bilateral Achilles tendon release. She was never able to climb stairs without holding on to the bannisters, subsequently losing the ability to climb stairs entirely by the age of twenty years of age. She became wheelchair-bound at the age of 48 years. She also developed proximal muscle weakness of the arm and diminished handgrip strength. She subsequently developed swallowing difficulties and symptoms of nocturnal hypoventilation around the age of 51 years.

Neurologic examination at the age of 51 revealed mild myopathic facies, high arched palate, extensive atrophy of arms and legs, neck extension weakness (MRC grade 4), weakness of deltoid (4/4), infraspinatus (MRC grade 4), triceps (MRC grade 4), wrist and hand muscles (MRC grade 3–4), psoas (MRC grade 4), hamstrings (MRC grade 4), quadriceps (MRC grade 3), gluteal (MRC grade 4), adductors (MRC grade 4), and feet dorsiflexors and plantar flexors (MRC grade 1). There was generalized areflexia. Sensation and coordination were intact.

Echocardiography revealed slightly reduced left ventricular ejection fraction. Forced vital capacity (FVC) was 2.8 L (68% of predicted) with an FEV1 of 60%. Serum CK (at the age of 51) was 289 U/L. Nerve conduction studies showed normal conduction velocities. Muscle MRI and muscle biopsy were not performed.

Sanger sequencing of *TPM3* identified the heterozygous c.8A > G (p.Glu3Gly) variant in *TPM3* that was previously identified in her son. Further genetic testing in her family was not possible. The patient passed away of lung cancer at the age of 53.

Patient 3, a 47-year-old Caucasian male, reported normal motor development. His symptoms started around the age of 6 years of age when he reported toe-walking and gradually falling behind his peers when running. He progressively became unable to run. At the age of 16 years, he underwent surgical correction of bilateral Achilles tendon contractures. Over the years he reported difficulty climbing up stairs and difficulty standing up from a squatting position. At the age of 40 years, he complained of weakness in his upper extremities (difficulties with lifting objects above his shoulders and reduced handgrip strength). Additionally, he complained of progressive exercise intolerance. At the age of 43 years, he required the use of a crutch and bilateral ankle foot orthoses to walk, subsequently requiring the use of a motorized wheelchair at the age of 47 years for ambulation. There was no involvement in swallowing and speech. He did not have any respiratory complaints.

The patient’s father, who is on follow-up in a different neurology center, was diagnosed with distal predominant nemaline myopathy. He has two younger sisters and one younger brother who remained well.

Neurological examination at the age of 40 years showed mild scapular winging, severe atrophy (distal greater than proximal) of arms and legs, toe clawing and Achilles tendon contractures bilaterally. Neck flexor weakness (MRC grade 4−), symmetric muscle weakness of shoulders (MRC grade 4−), elbow extension (MRC grade 4+), elbow flexion (MRC grade 4−), wrist and intrinsic finger muscle strength (MRC grade 3), hip flexion and extension (MRC grade 3), knee flexion (MRC grade 4+), knee extension (MRC grade 3), ankle dorsiflexors (MRC grade 3), and ankle plantar flexors (MRC grade 4−). Muscle tone was normal and deep tendon reflexes were absent. Sensation and coordination were intact.

Echocardiography performed at age 41 years was within normal limits. FVC was 3.47 L (78% of predicted) with an FEV1 of 2.81 L (76% of predicted). Serum CK level was 386 U/L. Nerve conduction studies were normal. Electromyography showed myopathic changes with no spontaneous activities or myotonic discharges.

**Figure 2:**
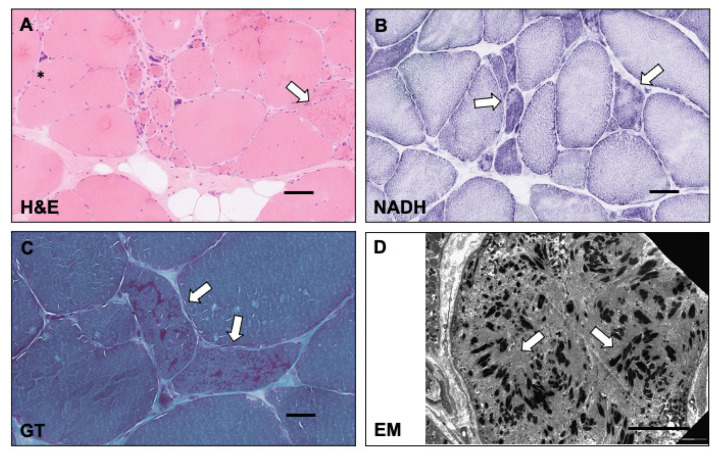
Muscle biopsy features. (**A**) H&E shows myopathic features with an increase in variation of fibre size, presence of small atrophic fibres, large hypertrophic fibres, increase in internal nuclei, nuclear bag fibres, split fibres (asterisk), intracytoplasmic inclusions (arrow), occasional vacuoles, endomysial fibrosis, and adipose tissue in the perimysium. (**B**) NADH histochemistry shows disrupted internal architecture, particularly in the smaller fibres with irregular and pale staining (arrows). (**C**) Rod-like inclusions are noted in a proportion of fibres, mainly the small fibres on the Gomori Trichrome stain. (**D**) Ultrastructural examination revealed rod-like structures (arrows) of varying orientation in a fibre lacking normal myofibrillar material. Scale bars: panels (**A**–**C**) 10 μm, panel (**D**) 5 μm.

Muscle biopsy showed a chronic myopathy with intracytoplasmic inclusions, occasional vacuoles, and nemaline rod inclusions mainly in small muscle fibres (features detailed in Figure 2).

A muscle MRI was not performed. An NGS myopathy panel was performed and identified the heterozygous c.8A > G (p.Glu3Gly) variant in the *TPM3* gene in both the patient as well as his father [15].

### 2.2. Functional Significance of the Amino Acid Substitution p.Glu3Gly in Tpm3.12

To understand the molecular bases of the observed clinical phenotypes in the patients, functional studies were performed. Effects of the mutation on Tpm3.12 polymerization, interaction with actin filament, and Ca^2+^-dependent regulation of actomyosin ATPase were analyzed. The biochemical assays were performed with the use of recombinant wild-type Tpm3.12 and the variant p.Glu3Gly (Tpm3.12-E3G).

#### 2.2.1. End-to-End Interactions of Tpm3.12 and Tpm3.12-E3G

**Figure 3:**
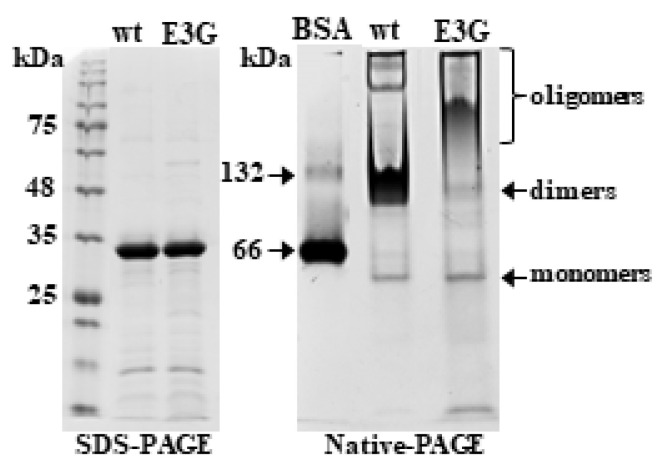
Tpm3.12 wild-type (wt) and mutant (E3G) separated on SDS-PAGE (**left panel**) and Native-PAGE (**right panel**). The molecular mass standard used on 12% SDS-PAGE was Perfect Tricolor Protein ladder (EURx). The molecular mass standard used on the 8% Native-PAGE was Bovine Serum Albumin Standard (Pierce). In both types of gels, each lane was loaded with 10 µg of protein.

Since the variant causes a substitution of an amino acid in the N-terminal segment of Tpm3.12, it can affect the interactions between the C- and N-terminal segments, which are involved in the overlap formation between Tpm molecules. To examine this possibility, we applied nondenaturing polyacrylamide gel electrophoresis. As illustrated in Figure 3, replacing the negatively charged Glu by the neutral Gly did not change the electrophoretic mobility of Tpm3.12 in the reducing SDS-PAGE (left panel), but it had a large effect on the mobility of Tpm3.12 in the reducing gel from which SDS was omitted (right panel). To distinguish between the coiled coil monomers (two folded Tpm3.12 chains) and dimers (two overlapping coiled coils), as a marker we used bovine serum albumin (BSA), which in solution exists mainly as a 66 kDa monomer, but also as a dimer with the molecular mass of about 132 kDa [16]. Although BSA is a globular protein and Tpm3.12 is rod-shaped, both have an equivalent molecular mass, therefore BSA was used to estimate localization of monomers, dimers, and higher oligomers on the native gel. The dominating species observed for wild-type Tpm3.12 were dimers with small contamination of monomers and higher oligomers. In contrast, the band of the Tpm3.12-E3G mutant was smeared, with the upper edge located much higher than that assigned to a dimer (Figure 3). Thus, substituting the acidic Glu with a neutral Gly residue increased the propensity of Tpm3.12 to form higher oligomers.

#### 2.2.2. Effects of the p.Glu3Gly Mutation in Tpm3.12 on the Thin Filament Assembly

**Figure 4:**
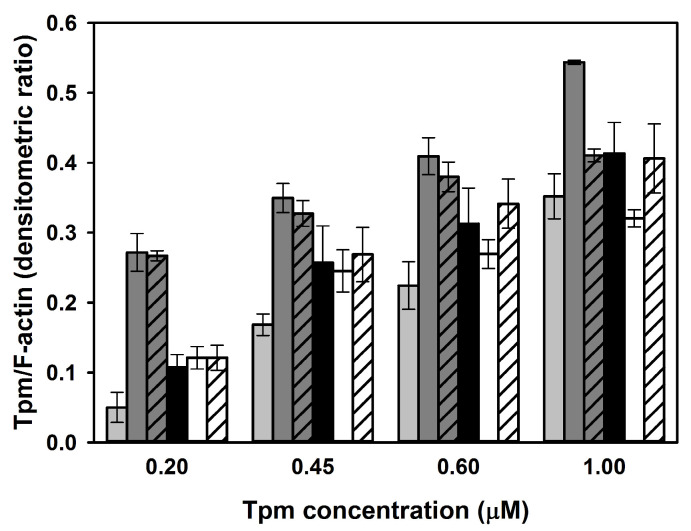
Effects of the substitution p.Glu3Gly on the thin filament assembly. Binding of Tpm3.12 and Tpm3.12-E3G to F-actin alone and in the presence of 1.5-molar excess of Tn, either with 0.1 mM CaCl2 or 0.2 mM EGTA. The bars are color-coded as follows: Tpm3.12 (light grey bars), Tpm3.12 + Tn + CaCl_2_ (dark grey bars), Tpm3.12 + Tn − CaCl_2_ (dark grey bars with diagonal lines), Tpm3.12-E3G (black bars), Tpm3.12-E3G in the presence of Tn + CaCl_2_ (white bars), Tpm3.12-E3G in the presence of Tn − CaCl_2_ (white bars with diagonal lines). Conditions: 3 μM F-actin, 30 mM NaCl, 2 mM MgCl2, 5 mM imidazole, pH 7.0, and 1 mM DTT. The bars show average values from three independent experiments ± SE.

As increased end-to-end interactions can facilitate assembly of Tpm chains on the thin filament [17], the effect of the substitution p.Glu3Gly on steady-state binding of Tpm3.12 to F-actin in the absence and presence of Tn was analyzed by a cosedimentation assay. To compare binding of Tpm3.12 and Tpm3.12-E3G, increasing concentrations of both forms of Tpm, alone or with 1.5-fold molar excess of Tn, were added to F-actin and ultracentrifuged. Proteins collected in pellets were separated on SDS-PAGE and quantitated by densitometry. Saturation of F-actin with Tpm was estimated as a densitometric ratio of Tpm and F-actin bands. As demonstrated in Figure 4, Tpm3.12-E3G tended to bind better to F-actin than Tpm3.12, however, the differences between the wild-type and mutant version were not statistically significant.

Better binding of the mutant Tpm3.12-E3G compared to wild-type Tpm3.12 was observed over a wide range of concentrations (Supplementary Figure S1A). Since Tpm3.12-E3G formed stronger end-to-end interactions (Figure 3), the higher Tpm/actin ratios obtained for Tpm3.12-E3G in pellets could be due to additional sedimentation of the Tpm3.12-E3G polymers that were not bound to actin. To test this possibility, Tpm3.12-E3G was ultracentrifuged in the absence of F-actin. Supernatants and pellets obtained for 2 μM wild-type and mutant Tpm3.12 were collected and analyzed using electrophoresis. As illustrated in Supplementary Figure S1B, traces of both Tpm3.12 variants were observed in pellets, indicating that Tpm3.12-E3G bound specifically to F-actin.

**Figure 5:**
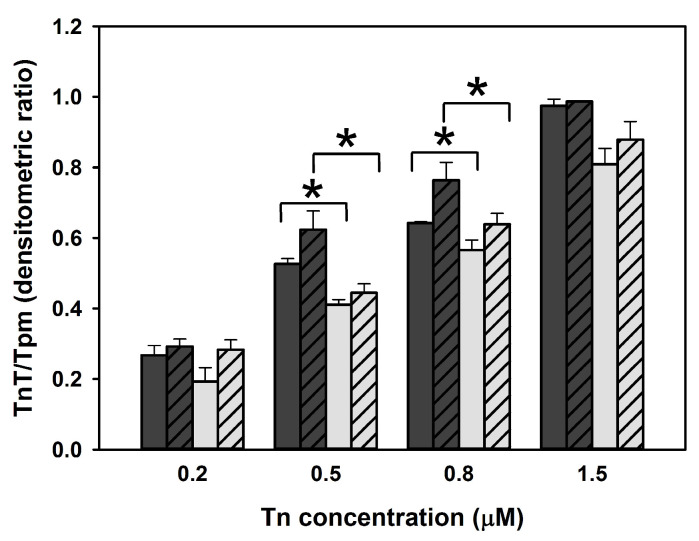
Binding of Tn to F-actin coated with Tpm3.12 (dark gray bars) and Tpm3.12-E3G (light gray bars) in the presence of 0.1 mM CaCl_2_ (empty boxes) or 0.2 mM EGTA (diagonal lines). * Statistically significant differences (*p* < 0.05) between the wild-type and mutant Tpm3.12 were observed at 0.5 μM and 0.8 μM Tn. Conditions: 3 µM F-actin, 1 µM Tpm3.12 or Tpm3.12-E3G, 30 mM NaCl, 2 mM MgCl_2_, 5 mM imidazole, pH 7.0, 1 mM DTT, and 0.1 mM CaCl_2_ or 0.2 mM EGTA. The bars show average values from three independent experiments ± SE. * statistically significant differences, *p* < 0.05.

In contrast, binding of Tpm3.12-E3G in the presence of Tn was impaired. Unlike wild-type Tpm3.12, the mutant Tpm3.12-E3G did not bind better to actin in the presence of Tn as opposed to in the absence of Tn, either with or without Ca^2+^. This might be caused by defects in binding of the Tn complex to the filament reconstituted in the presence of Tpm3.12-E3G. To test this possibility, a cosedimentation assay was performed, but this time the analysis was performed with progressively increasing Tn concentrations together with a constant F-actin-Tpm. As indicated by the analysis of changes in the densitometric TnT/Tpm ratio, the substitution p.Glu3Gly decreased interactions between Tn and Tpm (Figure 5), which confirms that the presence of Tpm3.12-E3G disturbed the association of Tn with the thin filament.

#### 2.2.3. Ca^2+^-Dependent Regulation of the Actin-Activated Myosin S1 ATPase by Wild-Type or Mutant Tpm3.12 and Tn Complex

In muscle, the main function of the Tpm–Tn complex is to regulate actin–myosin interactions in response to changing levels of the intracellular Ca^2+^. In light of this, we applied functional in vitro assays in which the activity of actin-activated myosin ATPase was measured. Soluble myosin S1, the globular subfragment comprising an actin-binding site and ATPase activity, was used.

**Figure 6:**
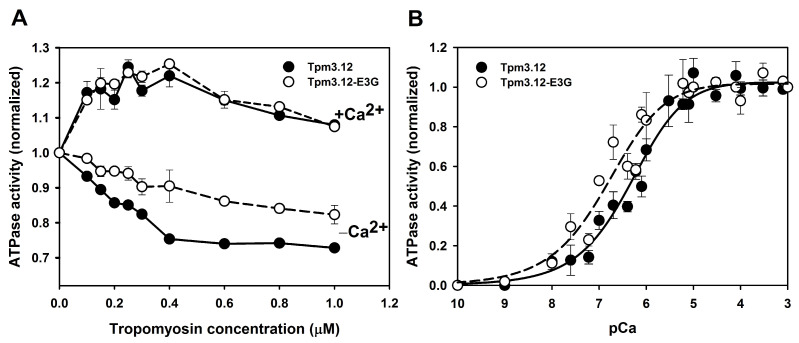
Regulation of actin-activated myosin S1 ATPase in the presence of Tpm3.12 (black circles) and Tpm3.12-E3G (white circles). (**A**) Activation (+0.1 mM CaCl_2_) and inhibition (0.2 mM EGTA) of the ATPase as a function of increasing concentrations of wild-type or mutant Tpm3.12 and Tn at 1.5-fold molar excess over Tpm. (**B**) ATPase activity measured as a function of Ca^2+^ concentration, at 1 μM Tpm and 1.5 μM Tn. Conditions: 5 μM F-actin, 1 μM S1, 30 mM NaCl, 2 mM MgCl_2_, 5 mM imidazole, pH 7.0, and 1 mM DTT. Each point is an average of three independent experiments ± SE.

In the first type of assay, ATPase activity was measured at increasing Tpm–Tn concentrations and constant concentrations of F-actin and myosin S1. In the presence of Ca^2+^, activation of the actomyosin ATPase was similar for the wild-type and mutant Tpm3.12. However, at low Ca^2+^ concentration, the presence of Tpm3.12-E3G on the filament significantly reduced inhibition of the ATPase (Figure 6A).

Next, we analyzed the dependence of the ATPase on increasing Ca^2+^ concentration. The Ca^2+^ titration curves obtained either in the presence of Tpm3.12 or Tpm3.12-E3G are shown in Supplementary Figure S2. Normalization of these data (Figure 6B) revealed that in the presence of Tpm3.12-E3G the activation curve was shifted toward lower Ca^2+^ concentrations, indicating that the sensitivity of the regulatory proteins to activating Ca^2+^ concentrations was increased. The pCa_50_ values, which were the pCa values at the inflexion points of the curves, were computed by fitting the experimental points to the Hill equation. For the wild-type Tpm3.12, pCa_50_ = 6.3 ± 0.02, and for the Tpm3.12-E3G, pCa_50_ = 6.7 ± 0.13. This translates to approximately 0.50 μM and 0.20 μM Ca^2+^ required to achieve 50% of activation in the presence of Tpm3.12 and Tpm3.12-E3G, respectively.

In summary, the analyses of the Ca^2+^-dependent regulation of actin–myosin interactions showed that the substitution p.Glu3Gly in Tpm3.12 impaired the ability of the thin filament regulatory complex to inhibit interactions of myosin with actin.

## 3. Discussion

Gathering functional evidence for the pathogenicity of a variant of unknown significance is essential for genetic counseling of the patient and his/her family, especially pertaining to reproductive options. When a variant can be classified as likely pathogenic or pathogenic, preimplantation genetic testing or prenatal testing (depending on local perspectives) may become an option for a patient.

This study reports a novel missense variant c.8A > G in *TPM3* that results in p.Glu3Gly. *TPM3* encodes the Tpm3.12 protein, a tropomyosin isoform specific for type 1 slow twitch muscle fibers. This variant is absent in gnomAD (version 3.1.2), and affects an amino acid that is conserved throughout vertebrates, in orthologue alignments, while lower species (fruit fly and nematodes) have another negatively-charged residue (Asp). The variant was found in three individuals from two unrelated families showing a similar clinical phenotype manifested by weakness of the limb muscles with concomitant contractures of the Achilles tendon. In vitro assays performed revealed that the substitution p.Glu3Gly affects Tpm3.12 polymerization and regulation of the thin filament, ultimately leading to increased actomyosin activation. Taken together, our findings allow us to classify the *TPM3* c.8A > G (p.Glu3Gly) variant as (likely) pathogenic.

### 3.1. Hypercontractile Phenotypes Linked to Mutations in TPM3 and TPM2

Most mutations in *TPM3* reported thus far have been associated with clinical and molecular hypocontractile phenotypes with muscle weakness due to reduced contraction activation and lower sensitivity to activating Ca^2+^ concentrations [7,8]. Reports on *TPM3* variants linked to the hypercontractile phenotype are limited. Donkervoort et al. [6] reported on two patients with in-frame intragenic deletions which resulted in p.Glu218del and p.Glu224del in Tpm3.12. Both patients presented with a severe generalized congenital muscle stiffness without muscle weakness. On the molecular level, both deletions showed increased force at submaximal Ca^2+^ concentrations in vitro, which most likely explained the clinical muscle stiffness. The molecular phenotype of the two deletion mutants resembled effects of the p.Glu3Gly variant that showed decreased inhibition of the actomyosin ATPase in the absence of Ca^2+^, and increased sensitivity to Ca^2+^ concentration. However, clinical phenotypes substantially differed, with p.Glu3Gly presenting with slowly progressive myopathy with limited contractures. A possible cause of these differences is the locus of the particular mutation. *TPM3* is a gene encoding several tropomyosin isoforms that are generated via selection of alternative exons [18,19]. Products of *TPM3* are tropomyosins that either associate with the contractile or with the cytoskeletal actin filaments. Tpm3.12 is a high molecular weight isoform with the N-terminal region encoded by alternative exon 1a. Cytoskeletal products of *TPM3* expressed in muscle are low molecular weight isoforms with the N-terminus encoded by exon 1b [18]. Thus, p.Glu3Gly is restricted to the high molecular weight tropomyosin present only in contractile thin filaments, and has no effect on cytoskeletal thin filament functions. In contrast, deletions p.Glu218del and p.Glu224del are in the region encoded by the constitutive exon 7, so that they affect both contractile and noncontractile thin filaments. By affecting various isoforms of tropomyosin, the mutations can disturb many different functions of actin filaments present in muscle cells, leading to a more severe clinical phenotype.

A mutation that is associated with a phenotype somewhat similar to the phenotype observed in our patients was found in *TPM2*, the gene encoding Tpm2.2 expressed in slow and fast twitch muscle fibers. The deletion p.Lys7del caused joint contractures with skeletal muscle weakness developing in adulthood [5,7]. Assays performed on patient myofibers and on recombinant mutant protein showed increased Ca^2+^ sensitivity of contraction [5]. Similar to p.Glu3Gly, the *TPM2* p.Lys7del variant is located within the end-to-end overlap protein region. Therefore, structural changes caused by both mutations may disturb interactions of Tpm with TnT, which can result in Ca^2+^-dependent regulation of actin–myosin interactions.

### 3.2. Mechanism of Releasing Inhibition of Actin–Myosin Interactions by Tpm3.12-E3G

Activation of the thin filament is described by the three-state model in which tropomyosin acts as a steric blocker [20]. In the relaxed state, Tpm chains are located on the outer edge of the thin filament blocking most of myosin binding sites on actin (blocked state). Binding of Ca^2+^ to TnC induces conformational changes, which releases the inhibition of actomyosin interactions by moving the C-terminal segment of TnI away from actin, and shifting Tpm chains towards the center of the filament (closed state). A further shift of Tpm is initiated by weak myosin binding (open or M state). An equilibrium between these structural states of the thin filament allows myosin heads to bind actin, and defines the “off” and “on” states of the actomyosin ATPase activity [12,13,21,22].

Increased Ca^2+^-sensitivity, along with impaired inhibition of the actin–myosin interactions, indicate destabilization of the functional “off” state. Since mutation-dependent changes in tropomyosin structure affect actin binding energy and shift tropomyosin azimuthally on the filament [23], effects of the mutations on the tropomyosin–actin interface can be considered as one of the mechanisms underlying the molecular phenotype. Marston and colleagues proposed that destabilization of the “off” state is due to substitutions or deletions of amino acids positioned downstream from the positively charged tropomyosin residues, which in the closed state make direct contact with negatively charged residues exposed on the actin surface [12,24]. In light of data collected in our present work, this model does not seem to be universal. The mutation studied in this work leads to a hypercontractile molecular phenotype, but it does not affect the tropomyosin–actin interface [14,25].

As mentioned above, localization of the mutation suggests that the variant p.Glu3Gly affects Tpm3.12 interactions with TnT. In the cosedimentation assay, Tpm3.12-E3G alone bound to actin as well or even better than the wild-type Tpm3.12, but Tn did not facilitate a further increase of Tpm3.12-E3G binding to actin, which was in contrast to the wild-type Tpm3.12. Thus, the variant p.Glu3Gly impaired interactions of Tn with the thin filament, which explains destabilization of the “off” state manifested by lower inhibition of the actomyosin ATPase. Thus, studies on the novel mutation provided evidence that the end-to-end overlap is not only important for polymerization of Tpm along the actin filament, but also for Ca^2+^-dependent regulation of contraction.

It had previously been proposed that the analysis of the energy changes due to alterations in the actin–tropomyosin interface allows one to predict the molecular phenotypes produced by mutations in tropomyosin [23]. Our data suggest that the additional factor determining the molecular phenotype in skeletal muscle is Tpm–Tn binding. Destabilization of interactions within the Tpm–Tn junction region can release inhibition by sensitizing the actomyosin interactions to Ca^2+^, leading to a hypercontractile phenotype.

### 3.3. Structural Determinants of the Molecular Phenotype Produced by the Substitution p.Glu3Gly

To date, a high-resolution structure of F-actin in complex with Tpm3.12 is not available. However, due to the highly homologous nature between the two molecules, structures obtained from Tpm1.1 can be used to explain possible mechanisms associated with Tpm3.12 at the molecular level. The N-terminal amino acid sequence of Tpm3.12 is almost identical to that of the fast skeletal Tpm1.1, except that Tpm3.12 has an additional N-terminal Met and Glu3 instead of Asp2 present in Tpm1.1. The NMR structures of the end-to-end overlap obtained for Tpm1.1 N- and C-terminal peptides [26] did not reveal any inter- or intrachain interactions of Asp2 in the overlap region. However, neutralization of the negative charge by substitution of Asp2 in Tpm1.1 or Glu3 in Tpm3.12 with Gly increases hydrophobicity of the N-terminal segment which can be the reason for stronger end-to-end interactions demonstrated by nondenaturing electrophoresis.

Although the Tn complex usually promotes binding of Tpm to actin [27], Tpm3.12-E3G bound similarly in the presence or absence of Tn, indicating that Tn interactions with the Tpm3.12-E3G-decorated filaments were impaired. This possibility was confirmed by the observation that the occupancy of the filament by the Tn complex at increasing concentrations of Tn was lower in the presence of Tpm3.12-E3G. Most likely, the variant affected the interactions between tropomyosin and TnT1, the N-terminal TnT segment that binds to the Tpm end-to-end overlap. Recent computational and 3D-EM models of the Tpm1.1-TnT junction obtained under relaxing conditions (at low Ca^2+^) predicted extensive interactions between the N-terminus of tropomyosin and TnT1 [14,28]. Although in these structures Asp2 did not form direct interactions, conformational changes within the end-to-end overlap region may affect binding of the entire Tn. TnT extends from the Tn core domain bound in the mid region of one tropomyosin chain to the tropomyosin–TnT1 junction of the second chain [12]. One can hypothesize that structural changes within the Tpm–TnT junction are transmitted across the filament to the Tn core region, and modify sensitivity to activating Ca^2+^ concentration.

Notably, recent in vitro studies of the functional effect of the p.Ala4Val mutation in Tpm3.12 revealed that, unlike p.Glu3Gly, it produced a hypocontractile molecular phenotype with lower activation of actomyosin ATPase activity in the presence of Ca^2+^ [29]. This shows that the structure of the N-terminal segment of tropomyosin is extremely important for the regulation of contraction. At this stage, it is not easy to predict the phenotype associated with each individual novel mutation. Therefore, in vitro studies are still necessary for the functional characterization of novel mutations. High-resolution structures of the thin filament containing wild-type and mutant Tpm3.12 variants in different activation states are required for full understanding of the molecular bases of Tpm-related disease.

### 3.4. Mechanisms of the Clinical Phenotype Associated with the Mutation p.Glu3Gly

Patients from both families carrying the c.8A > G (p.Glu3Gly) variant presented with similar clinical phenotypes—slowly progressive muscle weakness and Achilles tendon contractures from early childhood. The molecular phenotype found in this work provides molecular explanation for the contractures, but does not offer a direct explanation of muscle weakness. The hypercontractile features observed in our in vitro ATPase assays were mild with Tpm3.12-E3G, showing rather lower ability to inhibit the actin–myosin interactions at subactivating Ca^2+^ concentration. A possible mechanism prompted by the results obtained from this work was that Tn did not saturate the filaments formed in the presence of Tpm3.12-E3G to the same level as in the presence of the wild-type variant. Some fraction of Tpm3.12-E3G could be Tn-free, resulting in hypercontractility at the molecular level. In muscle, prolonged contractions can cause muscle wasting, as evidenced by fatty infiltrations seen in MRI, and the presence of nemaline bodies in the muscle histopathology.

Another possibility is that the hypercontractile molecular phenotype was obtained for homodimers of Tpm3.12 variants, whereas in slow muscle fibers, Tpm3.12 forms both homodimers and heterodimers with Tpm2.2 [30]. Comparative in vitro studies revealed that effects of various mutations may be different depending on whether they are present in homo- or heterodimers [31,32,33]. On the other hand, in patients with p.Met9Arg mutation in *TPM3*, the expression level of Tpm2.2 was strongly decreased, which resulted in the dominance of Tpm3.12 homodimers [34]. Therefore, the use of homodimers as in vitro models of Tpm-dependent myopathy is at least partially justified.

Fast and slow skeletal muscle fibers express functionally different Tn that are thought to be responsible for fine tuning the contractile response of different types of muscle to Ca^2+^ (recently reviewed in: [35,36]). Thin filaments that were used in this study were reconstituted with fast skeletal muscle Tn isoform. As the presence of slow skeletal Tn renders muscle fibers more sensitive to Ca^2+^ concentration [37], effects of the p.Glu3Gly variant in the presence of slow Tn isoform may be even more pronounced. Further functional studies are required to verify the proposed mechanisms of muscle weakness resulting from the *TPM3* c.8A > G (p.Glu3Gly) variant.

## 4. Materials and Methods

### 4.1. Patient Recruitment and Sample Collection

Two unrelated probands (one from The Netherlands and one from the United Kingdom) and one family member were identified by their local neurologist and clinical geneticist. Medical history was obtained and clinical evaluations were performed as standard neurologic evaluation and care. Muscle strength was graded based on medical research council grading (MRC). Written informed consent for publication was obtained from each family. Diagnostic tests, including muscle biopsies and MRIs, were obtained as part of regular diagnostic testing.

### 4.2. Genetic Testing

In patient 1, exome sequencing was performed as previously described [38]. In brief, an Agilent SureSelect Human All Exon 50 Mb kit (Santa Clara, CA, USA) was used to capture and enrich exons. Sequencing was performed using an Illumina HiSeq 2000 (San Diego, CA, USA) machine. Read mapping and variant calling were performed using BWA and GATK, respectively. A bioinformatic filter for a ‘muscle disorders’ gene panel was applied. This panel (version DG2.11) consisted of 150 genes implicated in various forms of myopathies, muscular dystrophies, myotonic syndromes, and myasthenic syndromes (https://www.radboudumc.nl/en/patient-care/patient-examinations/exome-sequencing-diagnostics/exomepanelspreviousversions/exomepanelspreviousversions/muscle-disorders, accessed on 5 November 2023). The detected variants were prioritized based on the following criteria: frequency in the population (<5% dbSNP, <5% Exome Aggregation Consortium (ExAC) database of >60,000 exomes, <1% in-house database of >5000 exomes), nucleotide and amino acid conservation (based on alignments), relation of the gene to disease (per family), and inheritance pattern. Patient 2 was analyzed by Sanger sequencing.

In patient 3, focused exome sequencing was performed using the SureSelect Focused Exome (Agilent, Santa Clara, CA, USA), according to the manufacturer’s protocol. Filtering of variants was performed using VarAft platform. We screened a list of 322 genes related to neuromuscular disease and hereditary cardiomyopathies reported in the Neuromuscular gene Table 2016 (http://www.musclegenetable.fr, accessed on 5 November 2023). First, we filtered out variants with an allele frequency higher than 1% in ExAC, 1000 Genome, or ESP6500 databases. We excluded all synonymous and deep intronic variants. Remaining variants were then classified using American College of Medical Genetics (ACMG) criteria, and those deemed likely or definitely pathogenic were confirmed using Sanger sequencing.

The variant c.8A > G in *TPM3* was deposited in ClinVar: National Center for Biotechnology Information. ClinVar; [VCV001709789.1], https://www.ncbi.nlm.nih.gov/clinvar/variation/1709789/ (accessed on 12 September 2023).

### 4.3. Protein Isolation, Expression, and Purification

Fast skeletal actin, myosin subfragment S1, and troponin complex were isolated and purified from New Zealand rabbit hind leg and back muscle, as described previously [39].

In order to prepare the slow muscle-specific Tpm3.12, cDNA translated into human Tpm3.12 sequence (NM_152263.4), with Ala-Ser N-terminal extension to compensate for the lack of N-terminal acetylation in bacterial cells [40], was synthesized and optimized for bacterial expression by Shanghai ShineGene Molecular Biotech, Inc. (Shanghai, China). The cDNA was cloned into pET11a vector (Novagen Inc., Madison, WI, USA). Wild-type cDNA construct was used as a template to prepare the mutant Tpm3.12 (Tpm3.12-E3G). The missense mutation was introduced using PCR-based oligonucleotide-directed mutagenesis kit (Agilent Technologies, Santa Clara, CA, USA) and the following forward oligonucleotide:

5′GAT CAT ATG GCT TCT ATG ATG GGC GCT ATC AAA AAA AAA ATG CAG3′

Codon that was mutated is underlined.

The oligonucleotide was synthesized and HPLC purified by Eurofins Genomics (Ebersberg, Germany). Plasmids were transformed into XL-1 supercompetent cells and, after DNA isolation (GeneMATRIX Plasmid Miniprep DNA Purification Kit, Eurx, Gdańsk, Poland), substitutions were verified by DNA sequencing in the Eurofins Genomics laboratory (Ebersberg, Germany).

Wild-type and mutant Tpm3.12 were expressed in *E. coli* BL21(DE3) cells (Novagen Inc., Affiliate of Merck KGaA, Darmstadt, Germany) and purified as described before [29,33]. Protein concentration was determined spectrophotometrically at 280 nm using molar extinction coefficient 17,880. The coefficient was calculated from the human amino acid sequence of Tpm3.12 in the web.expasy.org/protparam/tool (accessed on 5 November 2023).

### 4.4. Native-PAGE

Purified recombinant wild-type and mutant Tpm3.12 were mixed in equal volumes with sample buffer (0.01% Bromophenol Blue and 40% glycerol in 125 mM Tris pH 6.8) and loaded onto 8% polyacrylamide gels. Gels were run in Tris/Glycine buffer, pH 8.3 in 4 °C at 30 V for 30 min, then at 40 V for 2 h, and finally at 50 V for 6–8 h. After electrophoresis, gels were stained with Coomassie brilliant blue G-250.

### 4.5. Actin-Binding Assay

Binding of the wild-type and mutant Tpm3.12 to F-actin was determined using a cosedimentation assay as described before [33]. Briefly, 3 µM F-actin was titrated with Tpm (from 0–3 µM in experiments without Tn and from 0–1 µM in the presence of Tn, which was maintained at 1.5-fold molar excess over Tpm) at room temperature in 30 mM NaCl, 2 mM MgCl_2_, 5 mM imidazole, and pH 7.0. Proteins were incubated for 30 min and pelleted by ultracentrifugation in a Beckman (Beckman Coulter, Inc., Brea, CA, USA) rotor 42.2 Ti for 1 h at 200,000× *g*. Pellets were separated on 10% or 12% (in the presence of Tn complex) SDS-PAGE. Proteins bands were quantitated densitometrically. The densitometric tropomyosin per actin or (tropomyosin + TnT) per actin ratios were calculated and used to draw bar plots.

### 4.6. Actomyosin ATPase

Measurements of the regulation of actomyosin S1 ATPase activity by wild-type and mutant Tpm3.12 were conducted in 96-well microplates as described before [39]. Ca^2+^-dependent regulation of the ATPase activity was analyzed at constant 5 μM F-actin and 1 μM myosin S1. Tropomyosin concentration varied between 0 and 1 μM, and Tn was at 1.5-molar excess over Tpm. The data obtained at each experiment were normalized by dividing the activity measured for each Tpm–Tn concentration by the activity of acto-S1 ATPase in the absence of Tpm–Tn.

Ca^2+^-sensitivity of the acto-S1 ATPase was measured at Ca^2+^ concentrations varying from 1 × 10^−10^ to 1 × 10^−3^ M. The following protein concentrations were used: 5 μM F-actin, 1 μM myosin S1, 1 μM Tpm, and 1.5 μM Tn. The experimental points were normalized according to the equation:

(A − A_min_)/(A_max_ − A_min_) (1)

where A = ATPase activity at a given point, A_min_ = ATPase activity at 1 × 10^−10^ M Ca^2+^, and A_max_ = ATPase activity at 1 × 10^−3^ M Ca^2+^. The values of pCa_50_ were obtained by fitting the experimental points in SigmaPlot 12.5 to the Hill equation:

v = (A_max_ × pCa)^αH^ × pCa_50_^αH^/(1 + pCa^αH^ × pCa_50_) (2)

Statistical analysis was carried out in SigmaPlot 12.5. One-way ANOVA was used to identify statistical significance of the differences between wild-type and mutant Tpm3.12.

## Data Availability

The variant c.8A > G in *TPM3* was deposited in ClinVar: National Center for Biotechnology Information. ClinVar; [VCV001709789.1], https://www.ncbi.nlm.nih.gov/clinvar/variation/1709789/ (accessed on 12 September 2023). The raw data are available upon request.

## Supplementary Materials

The following supporting information can be downloaded at: https://www.mdpi.com/article/10.3390/ijms242216147/s1.
