# Supplementary material for: A Novel Variant in TPM3 Causing Muscle Weakness and Concomitant Hypercontractile Phenotype
Source: Int J Mol Sci. 2023 Nov 9;24(22):16147. doi: 10.3390/ijms242216147 (PMC10671854; doi:10.3390/ijms242216147)
Supplement: Supplementary file 1 [file ijms-24-16147-s001.zip › ijms-2686833-SM.pdf]

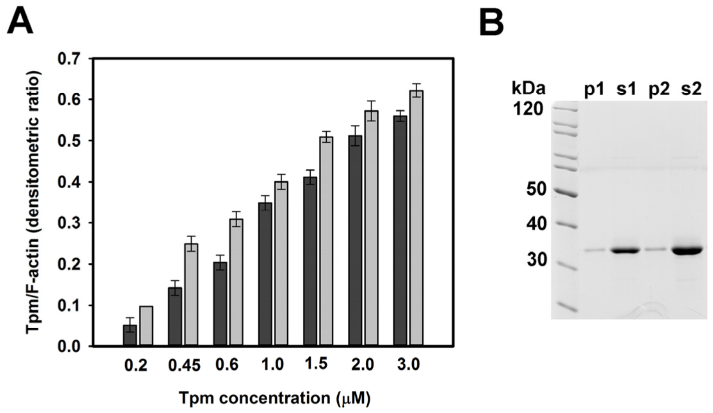

Supplementary Figure S1. A) F-actin saturation with Tpm3.12 (dark grey bars) and Tpm3.12-E3G (light grey bars). Conditions of the experiments: 3 μM F-actin, 30 mM NaCl, 2 mM $MgCl_2$, 5 mM imidazole, pH 7.0, 1 mM DTT. The bars show average values from three independent experiments ± SE. B) Pellets and supernatants after sedimentation of 2 μM Tpm3.12 (p1, s1) and Tpm3.12-E3G (p2, s2) in 40,000 rpm per 1 hour at 4°C.

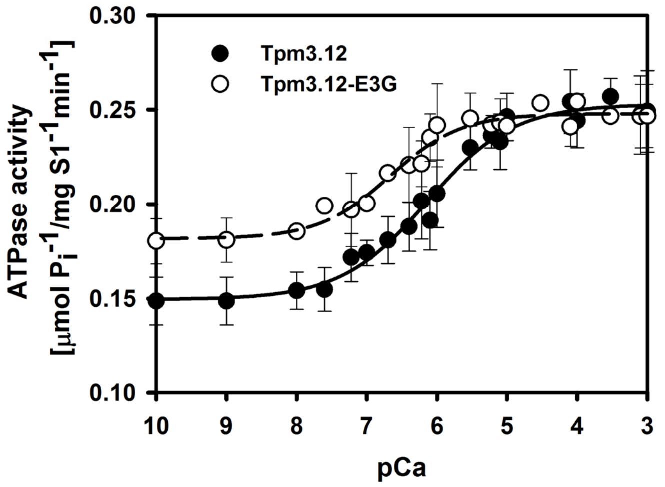

Supplementary Figure S2. ATPase activity measured as a function of Ca^{2+} concentration, at 1 μM Tpm and 1.5 μM Tn. Each point is an average of three independent experiments ±SE. Conditions: 5 μM F-actin, 1 μM S1, 30 mM NaCl, 2 mM $MgCl_2$, 5 mM imidazole, pH 7.0, 1 mM DTT.
